# Detection of the HA-33 protein in botulinum neurotoxin type G complex by mass spectrometry

**DOI:** 10.1186/s12866-015-0567-5

**Published:** 2015-10-23

**Authors:** Suzanne R. Kalb, Jakub Baudys, John R. Barr

**Affiliations:** Centers for Disease Control and Prevention, National Center for Environmental Health, Division of Laboratory Sciences, 4770 Buford Hwy, NE, Atlanta, GA 30341 USA

**Keywords:** Botulinum neurotoxin, Botulism, Mass spectrometry

## Abstract

**Background:**

The disease botulism is caused by intoxication with botulinum neurotoxins (BoNTs), extremely toxic proteins which cause paralysis. This neurotoxin is produced by some members of the *Clostridium botulinum* and closely related species, and is produced as a protein complex consisting of the neurotoxin and neurotoxin-associated proteins (NAPs). There are seven known serotypes of BoNT, A-G, and the composition of the NAPs can differ between these serotypes. It was previously published that the BoNT/G complex consisted of BoNT/G, nontoxic-nonhemagglutinin (NTNH), Hemagglutinin 70 (HA-70), and HA-17, but that HA-33, a component of the protein complex of other serotypes of BoNT, was not found.

**Methods:**

Components of the BoNT/G complex were first separated by SDS-PAGE, and bands corresponding to components of the complex were digested and analyzed by LC-MS/MS.

**Results:**

Gel bands were identified with sequence coverages of 91 % for BoNT/G, 91 % for NTNH, 89 % for HA-70, and 88 % for HA-17. Notably, one gel band was also clearly identified as HA-33 with 93 % sequence coverage.

**Conclusions:**

The BoNT/G complex consists of BoNT/G, NTNH, HA-70, HA-17, and HA-33. These proteins form the progenitor form of BoNT/G, similar to all other HA positive progenitor toxin complexes.

## Background

Botulism is a disease which can be fatal if untreated and is caused by intoxication with any of the extremely toxic proteins known as botulinum neurotoxins (BoNTs). BoNTs consist of a heavy chain, which binds to receptors on the neuron, and a light chain which serves as a protease, cleaving proteins necessary for nerve signal transmission. This enzymatic cleavage leads to flaccid paralysis, which can then lead to death if untreated. Botulinum neurotoxins are currently classified into seven confirmed serotypes, labeled A-G. BoNT/A, /C, and /E cleave SNAP-25 (synaptosomal-associated protein) [1–6] whereas BoNT/B, /D, /F, and /G cleave synaptobrevin-2 (also known as VAMP-2) [7–12]. BoNT/C is also known to cleave syntaxin [13, 14].

BoNTs are produced by *Clostridum botulinum*, *C. butyricum*, *C. baratii*, and *C. argentinese,* and are produced as a protein complex also known as the progenitor toxin, consisting of the neurotoxin and neurotoxin-associated proteins (NAPs). The composition of this complex can differ between serotypes, and in some cases, can differ within a serotype. For instance, the complex of BoNT/A1 Hall strain is reported to contain BoNT/A, NTNH, HA-70, HA-33, and HA-17 and is therefore hemagglutinin positive [15, 16], whereas the complex of BoNT/A2 is reported to contain only BoNT/A and NTNH [17] as the hemagglutinin proteins are not present, yet its genome contains open reading frames encoding for three proteins with controversial existence within the progenitor toxin [15, 17–19]. The role of these NAPs has not been completely deduced; however, it is likely that the NAPs serve to protect the progenitor toxin from harsh conditions found in the stomach, including low pH and digestive enzymes [20]. Additionally, it has been proposed that these NAPs assist with translocation of the neurotoxin across the intestinal epithelium [21], and the NAPs may assist with the immunogenicity of BoNT/A [22].

Characterization of the composition of the progenitor toxin of botulinum neurotoxins has been an area of abundant publication; however, characterization of the progenitor toxin of BoNT/G has been minimal. In 1991, it was reported that BoNT/G complex components separated by SDS-PAGE into 6 bands, with molecular masses of 150,000, 140,000, 58,000, 10,800, 10,600, and 10,400 [23]. Genetic analysis of the *Clostridium botulinum* type G toxin complex revealed the presence of genes for the neurotoxin, hemagglutinin, and nontoxin nonhemagglutinin [24], with further analysis defining these components as the neurotoxin, NTNH, HA-70, and HA-17 [25] and later including HA-33 [26]. Protein characterization of the progenitor complex of type G by mass spectrometry also revealed the presence of BoNT/G, NTNH, HA-70, and HA-17 [15, 27]. However, it was noted in the most recent publication [27] that the identity of one of the gel bands could not be determined. In this work, we show that the identity of that gel band is the protein HA-33, with identification by mass spectrometry including sequence coverage of greater than 90 %.

## Methods

### Materials

Botulinum neurotoxin is highly toxic and requires appropriate safety measures. All neurotoxins were handled in a class 2 biosafety cabinet equipped with HEPA filters. Commercially purified BoNT/G complex toxin was purchased (Metabiologics, Madison, WI). Sequencing-grade modified trypsin at 0.5 mg/mL in 50 mM acetic acid and sequencing grade chymotrypsin at 1 μg/μL in 50 mM ammonium bicarbonate was purchased (Roche, Pleasanton, CA). All chemicals were from Sigma-Aldrich (St. Louis, MO) except where indicated.

### Gel electrophoresis and digestion

SDS-PAGE gel electrophoresis was performed on a NuPAGE Novex Bis-Tris gel using the manufacturer-provided procedure (Invitrogen, Carlsbad, CA). Briefly, 1 μL of BoNT/G toxin complex at a concentration of 1 mg/mL was mixed with 2.5 μL of 4X sample buffer and 6.5 μL of deionized water and heated to 70 °C for 10 min. The mixture was then loaded on a 4-12 % gradient gel. The gel was stained with a Silver Stain kit (Protea Biosciences, Morgantown, WV) following the manufacturer-provided protocol. Selected gel bands containing visible stained protein were sliced and were destained by adding 10 μL of the Protea silver destaining solution and incubating for 30 min at room temperature, so that each band was fully destained. Each band was washed 3 times with 400 μL of water and once with 400 μL of 50 mM ammonium bicarbonate and then the protein in each band was reduced with 10 mM dithiothreitol at 60 °C for 30 min and alkylated with 55 mM iodoacetamide at room temperature in the dark for 30 min. The in-gel digestion was performed in 20 μL of 50 mM ammonium bicarbonate containing 0.2 μg of trypsin at 37 °C overnight. Following removal of the liquid, the gel band was then digested in 20 μL of 50 mM ammonium bicarbonate containing 0.2 μg of chymotrypsin at 37 °C overnight.

### LC-MS/MS analysis

NanoESI LC-MS/MS qualitative analysis was performed on an LTQ-Orbitrap Elite mass spectrometer (Thermo Scientific) connected to a nano-Acquity ultraperformance liquid chromatograph (UPLC; Waters, Milford, MA). 4 μL of the protein digest was injected on a C18 column (100 μm x 100 mm, 1.7 μm, BEH130 Å) and the peptides were separated using a linear gradient of 5-35 % of buffer B (acetonitrile, 0.1 % formic acid) over 80 min at a flow rate of 0.5 μL/min. The data acquisition was performed on an Xcalibur system using the data-dependent mode where the 15 highest-intensity precursors in an MS1 survey scan were isolated for collision-induced dissociation. The resulting MS/MS data were searched for protein candidates with a database search against an in-house BoNT database using MASCOT software (Matrix Sciences, London). The mass tolerance of precursor ions and fragment ions was 10 ppm and 0.8 Da, respectively. Resulting peptides were filtered with a significance threshold of *p* < 0.05 and an ion score cutoff of 40. The peptides with ion scores of less than 50 were validated by manual inspection. Quantitative analysis was performed as previously described [28], spiking the digests with a yeast alcohol dehydrogenase standard digest and using MS^E^ on a Synapt hybrid tandem mass spectrometer (Waters, Milford, MA).

## Results

**Fig. 1:**
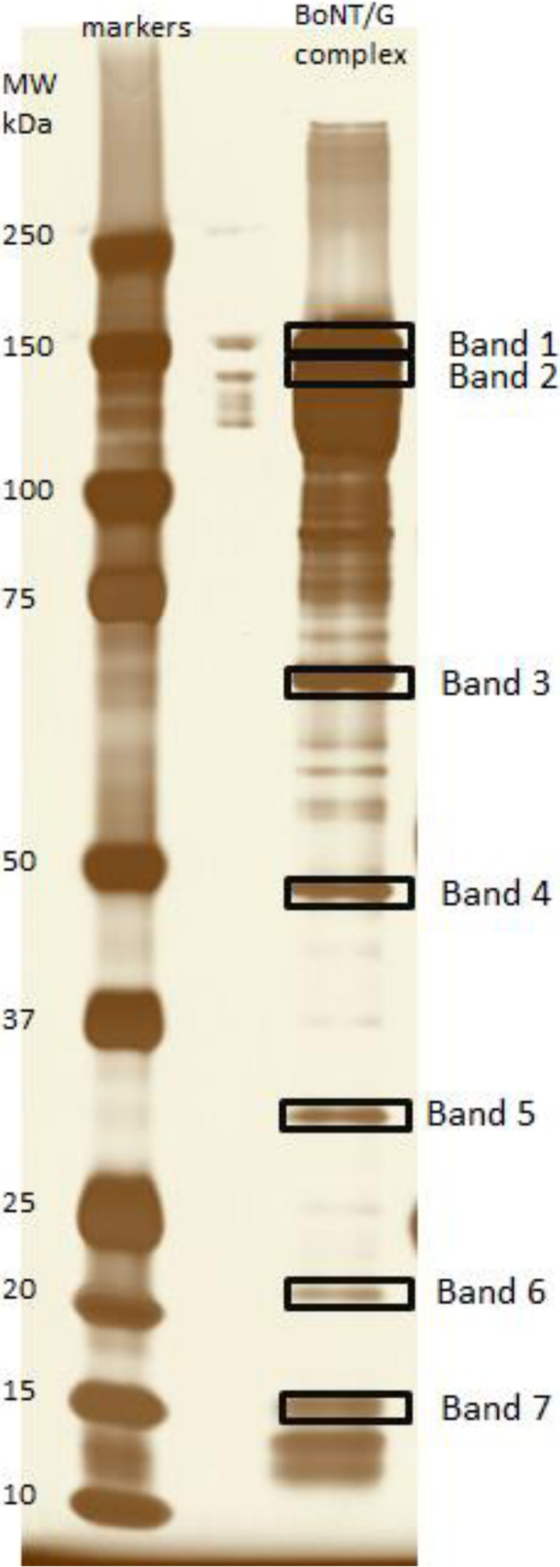
Silver stained SDS-PAGE of separated protein toxin components of BoNT/G. The first lane is molecular weight markers, the second lane is blank, and the third lane is the separation of the components of the BoNT/G complex. Seven bands as marked were excised, digested, and analyzed by LC-MS/MS with identities determined of band 1 as BoNT/G, band 2 as NTNH, band 3 as HA-70, band 4 as HA-70, band 5 as HA-33, band 6 as HA-70, and band 7 as HA-17

**Fig. 2:**
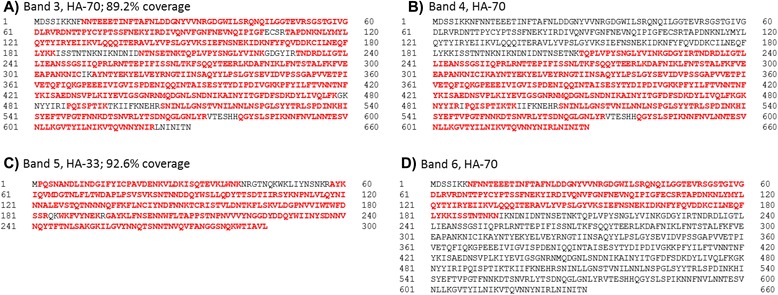
Amino acid sequence and sequence coverages of selected identified components of the BoNT/G protein complex. Residues for which MS/MS evidence exists are marked in red. **a** Band 3, identified as HA-70; **b** Band 4 identified as a C-terminal portion of HA-70; **c** Band 5 identified as HA-33; and **d** Band 6 identified as an N-terminal portion of HA-70

Separation of the BoNT/G complex by SDS-PAGE resulted in the presence of several bands (Fig. 1). Seven of the bands were excised, digested, and analyzed in a qualitative fashion by LC-MS/MS to discern the identity of the bands. The identities of the proteins in the gel are listed in Table 1, with their NCBI accession numbers and average masses. Band 1 was identified as BoNT/G with 90.8 % sequence coverage. Band 2 was identified as NTNH, also with 90.8 % sequence coverage. Band 3 was identified as HA-70 with 89.2 % coverage as seen in Fig. 2. It should be noted that the amino acid sequence is different from the HA-70 protein sequence identified in a previous publication [27].

**Table 1:** Components of the progenitor BoNT/G toxin complex

Protein description	Accession #	Avg mass (kDa)
BoNT/G	CA52275	149034
NTNH	CA61228	139083
HA-70	YP001893656	71742
HA-33	WP039635745	32822
HA-17	CAA61226	17372

The proteins identified in the BoNT/G complex, NCBI accession numbers, and average masses are listed

Band 4 was identified as HA-70 as well, but only a C-terminal portion consisting of approximately 2/3 of the total protein (Fig. 2). Band 5 was identified as HA-33 with 92.6 % sequence coverage (Fig. 2). Band 6 was identified as HA-70, but only an N-terminal portion of approximately 1/3 of the total protein (Fig. 2). Band 7 was identified as HA-17 with 87.8 % sequence coverage. Bands in the 75–100 kDa region were identified as portions of NTNH and BoNT/G and bands below 15 kDa were recognized as portions of HA-17 and NTNH. Quantification of bands 3, 4, and 6 yielded a ratio of band 3: band 4: band 6 as 1:7:3, whereas quantification of bands 5 and 7 yielded a ratio of 5:1 of band 5 to band 7.

## Discussion

The protein HA-33 is reported to exist as a part of the progenitor toxin complex for some strains of BoNT/A, /B, /C and /D [15]. In fact, the literature reports that for every botulinum neurorotoxin which contains HA-70 and HA-17 as part of its toxin complex, HA-33 is also present, with the exception of BoNT/G [15, 27]. Therefore, it should not be surprising that HA-33 is also present as part of the complex of BoNT/G, as we now understand that in every BoNT complex where HA-70 and HA-17 are present, HA-33 is also present. It should be noted that the gel separation in this work agreed with gel separations previously published [27]. The gel band which corresponds to HA-33 was previously reported as unidentified due to the absence of identifiable peptides following digestion and analysis [27]. Quantification of the proteins in the BoNT/G progenitor toxin complex shows that the amount of HA-33 is above the level of HA-17, and HA-17 was previously identified as part of the BoNT/G progenitor toxin complex, so it is likely that HA-70 was not previously identified due to the use of instrumentation with decreased sensitivity or the absence of HA-70 proteins from the database.

In this work, we also provide a longer amino acid sequence associated with the protein HA-70 than previously reported, giving this protein a molecular weight of 71742 Da. A previous report listed the identity of this gel band as a protein with only 488 amino acids, and a molecular weight of 55792 Da, likely due to the use of older instrumentation and database. This longer amino acid sequence more accurately reflects the identity of the protein as the protein elutes on a gel with an approximate molecular weight of 70 kDa. The amino acid sequence previously linked to this gel band only had a molecular weight of 52716 Da [27]. Two additional gel bands contained components of HA-70 with one composed of a C-terminal portion of approximately 2/3 of the intact HA-70 protein (Fig. 2) and the other an N-terminal portion of the remaining approximately 1/3 of the intact HA-70 protein (Fig. 2). Quantification of the bands shows that the most of the HA-70 proteins is present as the processed form rather than the intact form.

This phenomenon is not unusual and has been reported for other progenitor complexes of BoNT; namely BoNT/A and /C [15]. The progenitor toxins of both BoNT/A and /C are reported to possess HAs with molecular weights of approximately 52–55 kDa and 20 kDa respectively [16, 29], and these HAs are reported as components originating from the intact HA-70 gene product with proteolysis after translation of the gene. Proteolysis of the intact HA-70 gene product in BoNT/A and /C is reported to occur around amino acid 200, with the HA-52/55 protein composed of the C-terminal portion of approximately 2/3 of the intact protein and the HA-20 protein composed of the N-terminal remainder. The proteolytic region of HA-70 is not visible in the crystal structure of HA-70 of BoNT/A, indicating that this region is exposed while complexed and degraded due to proteolysis [30], and from these data here, it is likely that the HA-70 of BoNT/G is similar. Additionally, it should be noted that the data presented here were obtained from purified toxin complex which may have a different composition than toxin complex without purification.

In addition to proteolysis of HA-70, there is also evidence for limited proteolysis of other proteins. Bands also appear in the gel in the region of 75–100 kDa and 10–15 kDa. Mass spectrometric amino acid sequencing determined that these bands consisted of shorter versions of BoNT/G and NTNH in the 75–100 kDa region and HA-17 and NTNH in the 10–15 kDa region. This event is not original to BoNT/G, as our laboratory has observed this process in gels of other BoNT (data not shown), and this phenomenon has also been reported in prior publications involving other HA positive BoNT progenitor complexes [16, 31].

## Conclusion

In conclusion, through this work, we have discovered that the protein HA-33 is part of the BoNT/G progenitor toxin complex, and therefore, the BoNT/G complex consists of BoNT/G, NTNH, HA-70, HA-17, and HA-33. Thus, all HA positive progenitor botulinum toxin complexes known to date have the same composition. Additionally, we have discovered that the amino acid sequence previously reported for the HA-70 protein of the BoNT/G complex was incomplete due to the proteolytic processing of the HA-70 protein. This proteolytic processing is consistent with the proteolytic processing of HA-70 previously reported for other BoNT complexes.

## Abbreviations

BoNT: Botulinum neurotoxin
NAP: Neurotoxin-associated protein
NTNH: Nontoxic-nonhemagglutinin
HA: Hemagglutinin
ADH: Alcohol dehydrogenase
SDS-PAGE: Sodium dodecyl sulfate polyacrylamide gel electrophoresis
ESI: Electrospray Ionization
LC: Liquid chromatography
UPLC: Ultra performance liquid chromatography
MS: Mass spectrometry
MS/MS: tandem mass spectrometry
